# Protection of primary cilia is an effective countermeasure against the impairment of osteoblast function induced by simulated microgravity

**DOI:** 10.1111/jcmm.17628

**Published:** 2022-12-13

**Authors:** Jing Liu, Fei‐Fan Leng, Yu‐Hai Gao, Wen‐Fang He, Ju‐Fang Wang, Cory J. Xian, Hui‐Ping Ma, Ke‐Ming Chen

**Affiliations:** ^1^ Fundamental Medical Science Research Laboratories, The 940th Hospital of Joint Logistic Support Force People's Liberation Army of China Lanzhou China; ^2^ Department of Bioengineering, School of Life Science and Engineering Lanzhou University of Technology Lanzhou China; ^3^ Gansu Key Laboratory of Space Radiobiology Institute of Modern Physics, Chinese Academy of Sciences Lanzhou China; ^4^ UniSA Clinical and Health Sciences University of South Australia Adelaide South Australia Australia; ^5^ Department of Pharmacy, The 940th Hospital of Joint Logistic Support Force People's Liberation Army of China Lanzhou China

**Keywords:** BMP‐2, microgravity, miRNA‐129‐3p, osteoblasts, osteogenic differentiation, primary cilia

## Abstract

The molecular mechanism for the microgravity‐induced decrease in bone formation remains unclear and there is a lack of effective specific preventative therapies. We recently reported that primary cilia of osteoblasts became shorter and even disappeared when the cells were exposed to random positioning machine (RPM)‐simulated microgravity and that the microgravity‐induced loss of osteogenic potential of osteoblasts could be attenuated when the resorption of primary cilia was prevented by treatment with 0.1 μM cytochalasin D. In the current study, it was further found that the loss of the osteogenic capacity of rat calvarial osteoblasts (ROBs) was associated with the inhibition of the BMP‐2/Smad1/5/8 signalling pathway, of which most of the signalling proteins including BMP‐2, BMPRII, Smad1/5/8 and p‐Smad1/5/8 were found localized to primary cilia. Accompanying the resorption of primary cilia following the cells being exposed to simulated microgravity, the expression levels of these signalling proteins were reduced significantly. Furthermore, the expression of miRNA‐129‐3p, a microRNA previously reported to control cilium biogenesis, was found to be reduced quickly and changed in a similar tendency with the length of primary cilia. Moreover, overexpression of miRNA‐129‐3p in ROBs significantly attenuated microgravity‐induced inhibition of BMP‐2 signalling and loss of osteogenic differentiation and mineralization. These results indicated the important role of miRNA‐129‐3p in microgravity‐induced resorption of primary cilia of osteoblasts and the potential of replenishing the miRNA‐129‐3p as an effective countermeasure against microgravity‐induced loss of primary cilia and impairment of osteoblast function.

## 1 INTRODUCTION

Despite the rapid development in space exploration and the space industry, there are still many associated health problems of astronauts and cosmonauts waiting to be resolved before the space travel dream can be realized. Gravitational unloading due to microgravity has brought many negative effects on the body, including bone and muscle loss, cardiovascular dysfunction, malfunction in the nervous system and impaired immune function.^1^, ^2^, ^3^ Bone is very sensitive to mechanical stimulation^4^, ^5^, ^6^ and microgravity in space causes a reduction in the mass of load‐bearing bone, which is difficult to recover after the astronauts return to earth.^7^, ^8^ It was reported that 92% of the crew members of the Mir Space Station and the International Space Station (ISS) experienced a minimum 5% loss in at least one skeletal site and over 40% experienced a 10% or greater loss in at least one skeletal site.^9^ Bone loss of astronauts exposed to a long‐term space mission was reported to be about 10‐fold greater than that observed in postmenopausal women on earth.^10^ This significant change can affect the performance and safety of crew members during extravehicular activities and put them at a higher risk of fragility fractures.

Bone loss in space or analogue environment on earth has been considered to be the result of microgravity‐induced impairment of osteoblast function and the subsequent upregulation of osteoclast‐mediated bone resorption. It was reported that the simulated microgravity inhibited osteoblastogenesis but increased adipocyte differentiation of human mesenchymal stem cells,^11^ suppressed osteoblast mineralization by decreasing expression of alkaline phosphatase, osteocalcin, type I collagen α1 and runt‐related transcription factor 2 (Runx‐2).^12^ While the increased production of reactive oxygen species (ROS) and/or down‐regulated currents of L‐type voltage sensitive calcium channel were suggested to be induced by microgravity and inhibited the activity of osteoblasts,^13^, ^14^ work by several research groups had found microgravity‐induced changes in bone morphogenetic protein 2 (BMP‐2) signalling, a major signalling pathway for bone formation. Dai et al. revealed that simulated microgravity reduced the responsiveness of Runx‐2 to BMP‐2.^15^ Qin et al. demonstrated that BMP‐2‐induced osteogenesis of C2C12 cells was markedly reduced in simulated microgravity, which was partially rescued by inhibiting the expression of miR‐494, a microRNA directly targeting BMPRII and RUNX‐2.^16^ Siamwala et al. proposed to prevent bone loss in microgravity by manipulating BMP signalling.^17^ However, the detailed mechanisms for the microgravity‐induced changes in the BMP signalling pathway are still elusive.

The primary cilium is a microtubule‐based structure protruding from the cell surface like an antenna on most mammalian cells. While it was first described over a century ago and long considered to be a vestigial organelle of little functional importance,^18^ primary cilium has now been found to be a signal hub with ciliary defects being tightly coupled to a variety of developmental abnormalities and postnatal disorders, including oral‐facial‐digital syndromes, Bardet‐Biedl syndromes and ossification disorders.^19^, ^20^ Recent studies have demonstrated that some pharmacological agents and extracellular environmental changes can alter the length of primary cilia,^21^, ^22^, ^23^ and mechanical stimuli can change its orientation and trigger its biochemical and transcriptional changes.^24^, ^25^, ^26^ Studies in animals with a specific conditional knockout of the primary cilia in osteoblasts have shown that primary cilia are necessary for bone development, and primary cilium knockout reduces loading‐induced bone formation.^27^, ^28^ We have reported that the primary cilia of rat calvarial osteoblasts became shorter quickly and even disappeared in the microgravity environment simulated by a random positioning machine, and this phenomenon was accompanied by a significant reduction in osteogenic differentiation and mineralization of the osteoblasts. Interestingly, when the microgravity‐induced shortening/absorption of primary cilia was prevented with treatment with cytochalasin D, an inhibitor of actin polymerization, the loss of osteogenic capacity of osteoblasts was also attenuated.^29^ These results inferred that the primary cilium may be a microgravity sensor and that keeping the normal structure and functions of primary cilia should be a potential way to prevent microgravity‐induced loss of osteoblast function.^30^

In the current study, BMP‐2/Smad1/5/8 signalling was found to be closely connected with primary cilia of osteoblasts and its activity was inhibited simultaneously with the microgravity‐induced shortening/absorption of primary cilia. The overexpression of miRNA‐129‐3p, a microRNA reported to promote cilium biogenesis, had the ability to counteract the adverse effects of microgravity on primary cilia and BMP‐2 signalling pathway as well as the osteogenic capacity of osteoblasts.

## 2 MATERIALS AND METHODS

### 2.1 Reagents

α‐MEM culture medium and trypsin were obtained from Gibco (Gibco). Foetal bovine serum (FBS) was the product of Biological Industries (Beit‐Haemek). Collagenase II was purchased from Solarbio Life Sciences. β‐glycerophosphate, dexamethasone and ASAP (ascorbic acid 2‐phosphate) were all from Sigma. Human recombinant BMP‐2 was purchased from Beijing Biosynthesis Biotechnology Co. All other chemicals were of analytical grade.

### 2.2 Isolation and culture of rat calvarial osteoblasts (ROBs)

Primary osteoblasts were isolated from the calvarias of newborn SD rats as reported previously.^31^ Briefly, neonatal rats (within 24 h) were obtained from the Animal Breeding Center, Gansu University of Traditional Chinese Medicine (Lanzhou, China). Animal experiments were approved by the Animal Ethics Committee of the 940th Hospital of Joint Logistics Support Force of PLA and were conducted according to NIH Guidelines for the Care and Use of Laboratory Animals. The calvarias were dissected aseptically, cleaned of adhering soft tissues, cut into small pieces, digested twice at 37°C with 0.25% trypsin, 10 min each time, and then digested five times at 37°C with 1 mg/ml collagenase II, 15 min each time. The released cells from the last three digestions were pooled in α‐MEM culture medium and filtered through a 75 μm cell sieve. The collected cells were resuspended in α‐MEM plus 10% heat‐inactivated FBS and plated in a 100‐mm dish at a 37°C humidified incubator with 5% CO_2_. After reaching confluence, the cells were subcultured in 50‐ml flasks. The first to the third passages of osteoblasts were used in the following experiments.

### 2.3 Cell treatment in random positioning machine (RPM)

When the ROBs grew to near confluence, the culture medium was replaced with a fresh medium without FBS to enhance ciliogenesis by serum starvation.^32^ After 12 h, the flasks with osteoblasts were filled with the same medium without leaving any void space and fixed on a 3‐dimension (3D) clinostat to expose the cells to microgravity as we described previously.^29^ Briefly, the 3D clinostat is a random positioning machine (RPM, SM‐31) provided by the National Space Science Center, Chinese Academy of Sciences, which was composed of a desktop RPM and a control console. The RPM consists of two independent rotating frames, with one frame being positioned inside the other, and they rotate separately and randomly in the range of 0–10 rpm with changes in the acceleration and direction at 3D. The culture flasks mounted at the centre of RPM experienced a state of simulated microgravity (SMG). The RPM was placed in a CO_2_ incubator and connected to the control console outside of the incubator. ROBs in the normal ground (NG) group were seeded in flasks filled with the same culture medium but statically placed in a CO_2_ incubator without an RPM. The cell treatments as described above were applied in all experiments except for alkaline phosphatase (ALP) histochemical staining and mineralized nodule staining experiments described below.

### 2.4 ALP histochemical staining and mineralized nodule staining

To induce osteogenic differentiation and mineralization of osteoblasts, the cells grown to 80% confluence were cultured in an osteogenic induction culture medium supplemented with 10% FBS, 10^−8^ M dexamethasone, 10 mM β‐glycerol phosphate and 50 mg/ml ascorbic acid. The medium was refreshed every 3 days. After 9 days of RPM treatment, the cells were washed with PBS and fixed in 4% paraformaldehyde for 5 min, and then stained for 15 min at 37°C in an ALP staining solution (Solarbio). After 12 days of the RPM treatment, mineralized nodules formed were stained with 1% Alizarin red at 37°C for 30 min. The areas of the colonies stained positive for ALP (CFU‐F‐ALP) and the mineralized nodules were measured with Image‐J (Media Cybernetics). The results were expressed as percentages of CFU‐F‐ALP or mineralized nodules in the total area of the flask bottom.

### 2.5 Immunofluorescence staining and measurement of primary cilia

ROBs were grown on coverslips and were fixed in 4% paraformaldehyde and then permeabilized with 0.2% Triton X‐100 (PBST) for 10 min. After a brief wash, they were incubated for 1 h in 1% bovine serum albumin in PBS. Immunofluorescent staining was carried out overnight at 4°C with primary antibodies against acetylated α‐tubulin, γ‐tubulin, BMP‐2, BMP receptors (BMPRII, BMPRIA and BMPRIB), Smad1/5/8 or phosphorylated (p)‐Smad1/5/8 (1:500, all from Abcam). After washes, the cells were probed with secondary antibodies conjugated with Alexa 594 or Alexa 488 (1:1000; Abcam) for 1 h at 37°C. Cellular nuclear DNA was stained with 4′‐6‐diamidino‐2‐phenylindole (DAPI) (1:800; Solarbio). The cells were imaged under Delta Vision Imaging System (Delta Vision Ultra; Cytiva). The lengths of primary cilia were measured with Image‐Pro Plus 6.0 software (Media Cybernetics). The average length of each group was calculated from at least 40 primary cilia (*n* = 40). The percentage of ciliated cells was obtained by counting the number of ciliated cells and the total number of cells in 10 different fields of view.

### 2.6 RNA interference of IFT88 and overexpression of miRNA‐129‐3p

To prevent ciliogenesis, ROBs were treated with a short hairpin RNA (shRNA) to knock down the expression of intraflagellar transport 88 (IFT88), a protein required for the formation and assembly of primary cilia. When the cells reached about 70% confluence, they were transfected with the shRNA targeting IFT88 (sequence: 5′‐GGAUAUGGGUCCAAGACAUCC‐3′) or a scrambled negative control shRNA using lipofectamine 2000 (Invitrogen).^29^ Transfection was indicated by the expression of green fluorescent protein (GFP) 24 h later. Gene silencing efficiency was evaluated after 48 h of transfection by RT‐PCR and Western blotting assays as described below. The cells with a high silencing efficiency were used to test their responsiveness to BMP‐2 underground gravity conditions.

Since a previous study reported that overexpression of miRNA‐129‐3p promotes cilia biogenesis,^32^ the present study investigated the effects of overexpression of this miRNA on primary cilia in ROBs. For overexpression of miRNA‐129‐3p, ROBs were transfected with scrambled negative control (NC) or miRNA‐129‐3p (sequence: 5′‐AAGCCCUUACCCCAAAAAGCAU‐3′) (synthesized by Gene Pharma Co. Ltd.) using Lipofectamine 2000. The transfection was indicated by green fluorescent protein (GFP) after 24 h. After 48 h incubation, the cells with a high transfection efficiency were used to measure changes in the length and incidence of primary cilia and their osteogenic potential under the RPM‐simulated microgravity.

### 2.7 Real‐time PCR analysis

Total cellular RNA was extracted using TaKaRa MiniBEST Universal RNA Extraction Kit (Takara Biotechnology), and single‐stranded cDNA was synthesized (Primescrip RT Reagent kit; Takara Biotechnology). Using specific primers for *IFT88*, *BMP‐2*, *BMPRII* and *miR‐129‐3p* designed from rat cDNA sequences and synthesized by Takara Biotechnology, mRNA expression levels of the genes were analysed by real‐time PCR on an ABI Biosystems 7300 (Applied Biosystems). All reactions were carried out in triplicate, and the data were analysed using the 2^−ΔΔ*C*t^ method. Glyceraldehyde phosphate dehydrogenase (GAPDH) was used as an internal control. The expression level of miRNA‐129‐3p was analysed similarly but with the Hairpin‐it™ miRNA RT‐PCR Kit (GenePharma). The primer sequences are given in Table 1.

**TABLE 1:** Primer sequences used for real‐time RT‐PCR analyses.

Gene	Forward primer (5′‐3′)	Reverse primer (5′‐3′)
IFT88	TCAGGCTATTGAGTGGCT	TCTCGCAGAACTGGGTAT
BMP‐2	ACCGTGCTCAGCTTCCATCAC	TTCCTGCATTTGTTCCCGAAA
BMPRII	TGCAGATGGACGCATGGAG	GAGCCAGACGGCAAGAGCTTA
miR‐129‐3p	AGTTCTTCAAGCCCTTACCCC	AATGGTTGTTCTCCACTCTCTCTC
GAPDH	GGCACAGTCAAGGCTGAGAATG	ATGGTGGTGAAGACGCCAGTA

### 2.8 Western blotting

Total cellular protein was extracted in RIPA lysis buffer (Solarbio) and obtained by centrifugation at 4°C for 15 min at 12,000 rpm, and protein concentrations were quantified by a BCA kit (Solarbio). The samples were separated by 10% SDS‐PAGE (Solarbio) and transferred to a PVDF membrane. After being blocked with 5% skimmed milk in PBS for 2 h, the membrane was incubated separately overnight with the following rabbit antibodies at 4°C: acetylated α‐tubulin, γ‐tubulin, α‐tubulin, collagen type 1 (COL‐1), osterix (OSX), BMP‐2, RUNX‐2, BMPRII, BMPRIA, BMPRIB, p‐Smad1/5/8, Smad1/5/8, IFT88 or GAPDH (all from Abcam, 1:1000). After probing with a secondary antibody (goat anti‐rabbit HRP‐conjugated IgG at 1:1000, Abcam), immunoreaction signals were visualized with the ECL chemiluminescence reagent (Solarbio).

### 2.9 Statistical analyses

Data were presented as mean ± SD. Statistical analysis was performed using SPSS 20.0. Statistical differences of data were analysed using Bonferroni modification of the Student's *t*‐test for two groups or one‐way anova after LSD post hoc test for multiple groups. *p* Values less than 0.05 are considered to be significantly different.

## 3 RESULTS

### 3.1 Microgravity‐induced loss of the osteogenic potential of ROBs was related to the decreased activity of the BMP‐2/Smad1/5/8 signalling pathway.

**FIGURE 1:**
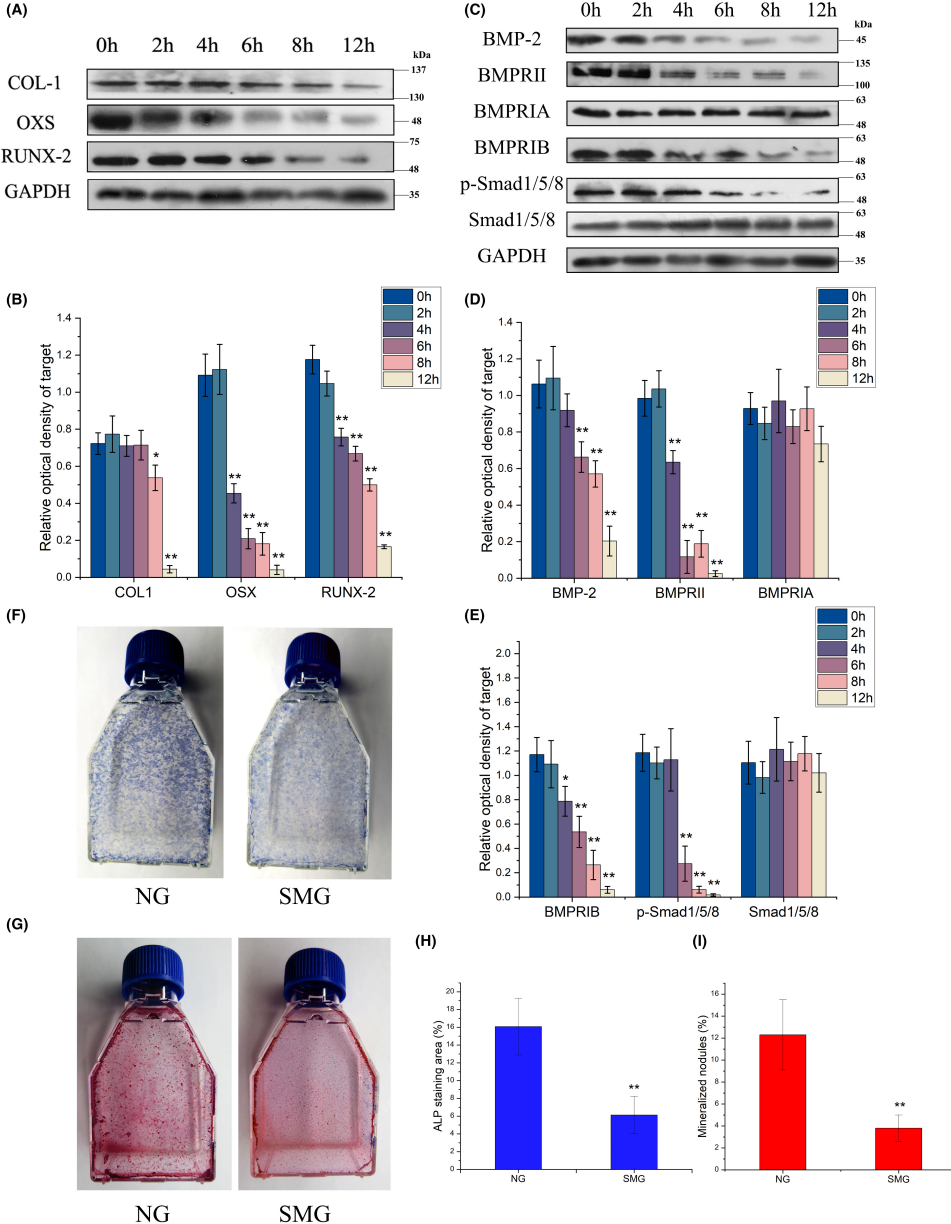
The changes of the osteogenic differentiation and mineralization of rat calvarial osteoblasts (ROBs) after the cells were exposed to the random positioning machine (RPM)‐induced microgravity for different periods. (A, B) Representative Western blots demonstrating levels of protein expression of osteogenic markers including COL‐1, OSX, and Runx‐2 with GADPH as an internal control. (C–E) Representative Western blots demonstrating expression levels of the signalling proteins of the BMP‐2/Smad1/5/8 pathway including BMP‐2, BMPRII, BMPRIA, BMPRIB, p‐Smad1/5/8 and Smad1/5/8. (F) Representative images of the alkaline phosphatase (ALP)‐stained CFU‐F‐ALP colonies after 9 days of RPM treatment. (G) Representative images of the Alizarin red‐stained mineralized nodules after 12 days of RPM treatment. (H) The areas of the CFU‐F‐ALP‐stained colonies. (I) The areas of the mineralized nodules. NG, normal gravity; SMG, simulated microgravity. Each experiment was conducted at least three times independently. Scale bar = 100 μm. *p < 0.05, **p < 0.01 vs 0 h (NG) group.

To investigate the effect of microgravity on osteoblastic differentiation and mineralization, osteoblasts grown to near confluence were treated by RPM for different periods, and then analysed for expression of COL‐1, OSX and RUNX‐2, the critical markers for osteogenic differentiation. It was found that the expression levels of these markers decreased significantly after 4 h (OSX and RUNX‐2) (*p* < 0.01) or 8 h (COL‐1) compared with 0 h (*p* < 0.05), and further decreased to a very low level after 12 h (Figure 1A,B) (*p* < 0.01), suggesting the significant reduction of osteogenic potential of osteoblasts after RPM treatment. To verify this changing tendency, ROBs were cultured in an osteogenic induction culture medium and treated by RPM, and numbers of colonies formed and stained positively for ALP (CFU‐F‐ALP**)** were analysed after 9 days, and numbers of calcified nodules formed were analysed after 12 days. As shown in Figure 1F–I, there were much fewer CFU‐F‐ALP colonies and calcified nodules in the simulated microgravity (SMG) group than in the normal gravity (NG) control group, which were confirmed by the quantitative results of the areas of CFU‐F‐ALP colonies and calcified nodules (Figure 1H,I).

To explore mechanisms for the decreased osteogenic potential of ROBs after RPM treatment, we investigated changes in the BMP‐2/Smad1/5/8 signalling pathway, a key pathway involved in osteoblastic differentiation. For this, the expression of proteins related to the pathway was examined by Western blotting in a time course, including BMP‐2, BMPRII, BMPRIA, BMPRIB, Smad1/ and p‐Smad1/5/8. Among these proteins, while only BMPRIA and Smad1/5/8 were kept constant in levels after different periods of RPM treatment, the other proteins all decreased significantly after 4 h (BMPRII) or 6 h (BMP‐2), and further decreased to a very low level after 12 h (Figure 1C–E), which were consistent with the changes in osteogenesis markers described above although they began to decrease earlier after RPM treatment. These results demonstrated that the decrease of the osteogenic potential of ROBs after RPM treatment was related to the inhibited status of the BMP‐2/Smad1/5/8 signalling pathway

### 3.2 The BMP‐2/Smad1/5/8 signalling pathway was closely connected with primary cilia and could not be activated in ROBs without primary cilia

**FIGURE 2:**
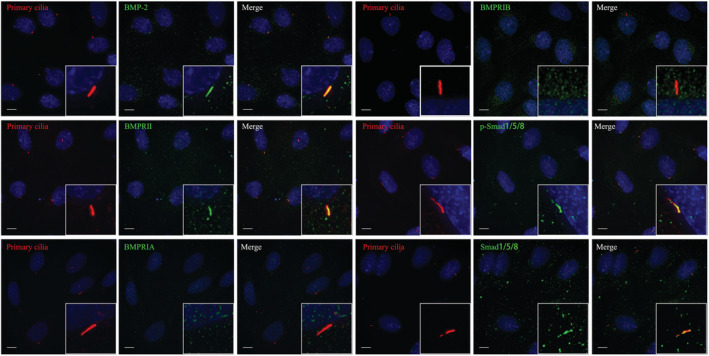
Co‐localization of the primary cilia of rat calvarial osteoblasts (ROBs) and the signalling proteins of the BMP‐2/Smad1/5/8 signalling pathway in normal gravity. Primary cilia were stained red with acetylated α‐tubulin, the signalling proteins were stained green, and the nuclei were stained blue with DAPI. Scale bar = 10 μm.

Since both primary cilia^29^, ^30^ and the BMP‐2/Smad1/5/8 signalling pathway (as described above) were significantly affected by simulated microgravity, we speculated that there exists a close relationship between the cilia and the pathway. To verify this speculation, we firstly examined whether one or more signalling proteins of the pathway were localized to primary cilia in the normal gravity condition. Surprisingly, it was found that immunofluorescence staining of BMP‐2, BMPRII, p‐Smad1/5/8 or Smad1/5/8 overlapped with primary cilia (Figure 2), in 84%, 90%, 93% and 85% of ROBs (Figure S1), respectively, indicating that these proteins were localized at the entire structure of a primary cilium. In addition, these proteins were all those whose expression levels were found to be decreased significantly after RPM treatment (Figure 1C–E). BMPRIA was not localized to primary cilia and its expression level was kept constant during RPM treatment. While BMPRIB was not localized to primary cilia either, its expression levels decreased significantly after RPM treatment.

**FIGURE 3:**
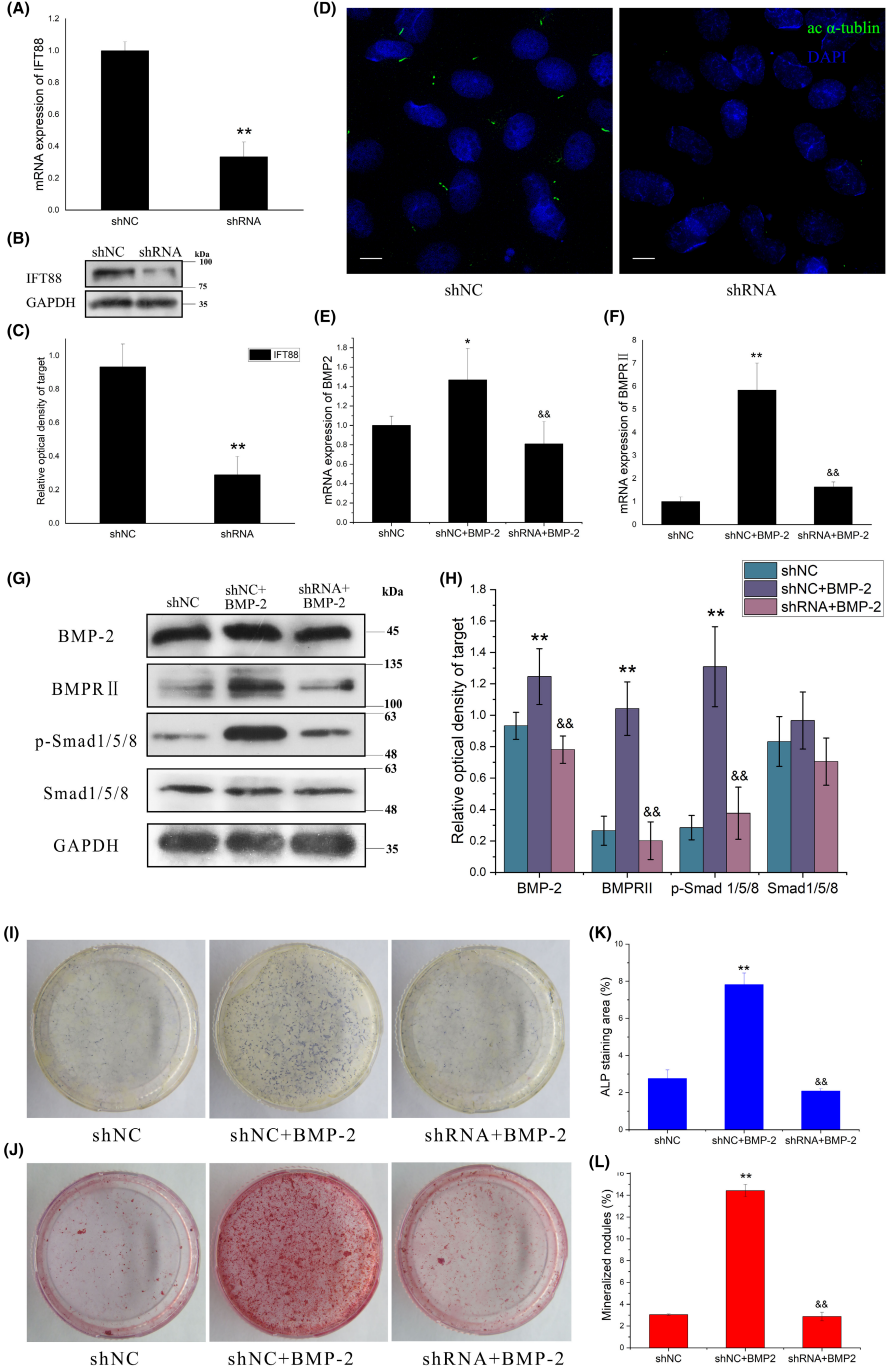
Effect of exogenous human recombinant BMP‐2 protein on the osteogenic differentiation and mineralization of rat calvarial osteoblasts (ROBs) with abrogation of primary cilia by shRNA silencing of IFT88 molecule (shRNA group). (A) The IFT88 mRNA expression level after 24 h and (B, C) IFT88 protein expression level after 48 h of transfection with shRNA targeting IFT88 in ROBs. (D) Primary cilia in the scrambled control group (shNC) and shRNA group. Primary cilia were stained green with acetylated α‐tubulin (ac α‐tubulin). (E, F) mRNA expression levels of osteogenic marker genes including BMP‐2 (E), and BMPRII (F) as revealed by real‐time RT‐PCR. (G, H) Representative Western blots reveal the effect of treatment with exogenous BMP‐2 on the expression levels of the BMP‐2 signalling proteins in the ROBs with or without primary cilia. (I) Representative images and the quantitative areas (K) of the alkaline phosphatase (ALP)‐stained CFU‐F‐ALP colonies after 9 days. (J) Representative images and the quantitative areas (L) of the Alizarin red‐stained mineralized nodules after 12 days. shNC: the scrambled control shRNA group; shRNA: the cilium‐abrogated group. Scale bar = 100 μm. *p < 0.05, **p < 0.01 vs shNC group. ^&&^ *p* < 0.01 vs shNC + BMP‐2 group.

The above results demonstrated a potentially close relationship between primary cilia and the BMP‐2/Smad1/5/8 signalling pathway. To explore the possibility that the decreased activation of the BMP‐2/Smad1/5/8 pathway was caused by defects of primary cilia, the primary cilia of ROBs were abrogated by silencing the expression of IFT88, a gene required for ciliogenesis. As shown in Figure 3A–D, primary cilia of ROBs were shorter and even became dotted after 24 h of transfection with shRNA against IFT88. We then examined the treatment effects on the BMP‐2/Smad1/5/8 pathway. While treatment with exogenous human recombinant BMP‐2 at 500 ng/ml significantly increased the mRNA expression levels of BMP‐2 and BMPRII in the normal control cells (shNC + BMP‐2), this increasing effect did not occur in the cells with abrogated primary cilia (shRNA + BMP‐2) (Figure 3E,F). While the expression levels of BMP‐2, BMPRII and p‐Smad1/5/8 were increased significantly in the shNC + BMP‐2 group compared with the shNC group, the expression levels of these proteins were not increased in the shRNA + BMP‐2 group and kept at similar levels with the shNC group (Figure 3G,H). Furthermore, the CFU‐F‐ALP colonies formed after 9 days (Figure 3I,K) and the calcified nodules formed after 12 days (Figure 3J,L) displayed similar tendencies among the three treatment groups. These results indicated that there is a close relationship between the primary cilia and the BMP‐2/Smad1/5/8 signalling pathway and that the pathway cannot be activated in osteoblasts without primary cilia.

### 3.3 Primary cilia were resorbed when osteoblasts were exposed to RPM‐induced microgravity

**FIGURE 4:**
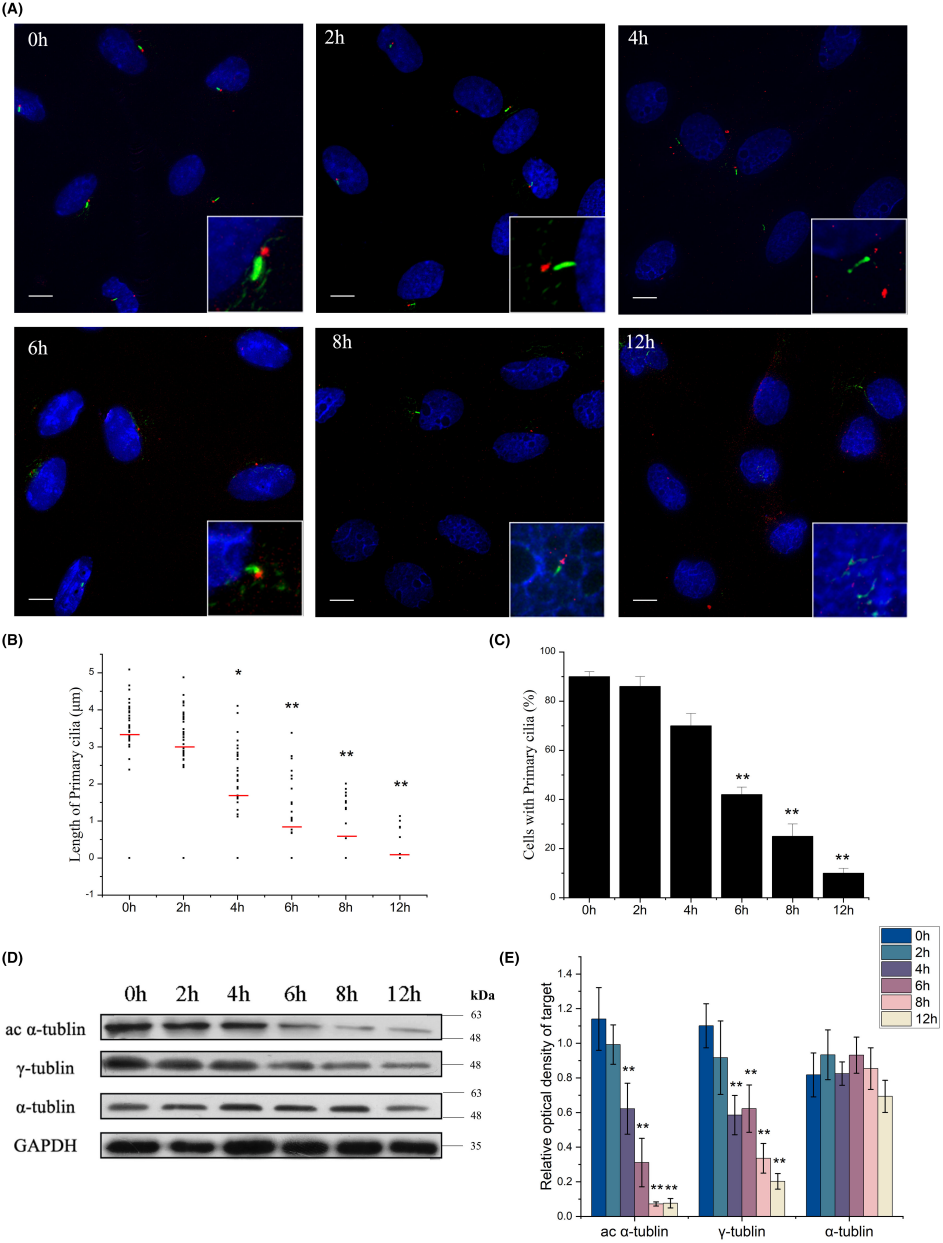
Changes in the length and incidence of the primary cilia of rat calvarial osteoblasts (ROBs) after the cells were exposed to the random positioning machine (RPM)‐induced microgravity for different periods. (A) The morphology of primary cilia after 4, 6, 8, and 12 h of exposure. The cilia were stained green with acetylated α‐tubulin and the basal bodies were stained red with γ‐tubulin. The nuclei were stained blue with DAPI. (B) The means (red bar) and individual measurements of cilium length. (C) The percent of the cells with primary cilia. (D, E) The protein expression levels of acetylated α‐tubulin (ac α‐tubulin), γ‐tubulin, and α‐tubulin. Each experiment was conducted at least three times independently. Scale bar = 10 μm. *p < 0.05 or **p < 0.01 vs 0 h.

To observe the effect of microgravity on the primary cilia of osteoblasts, ROBs were exposed to the RPM‐induced microgravity for 0, 2, 4, 6, 8 and 12 h, respectively, and were observed under the Delta Vision Imaging System after immunofluorescence staining with acetylated α‐tubulin and γ‐tubulin. It was found that the length of primary cilia and the number of the cells with primary cilia decreased quickly and depending upon the duration of microgravity exposure (Figure 4A). The average length of primary cilia was originally 3.2 μm (0 h), decreased by half after 4 h and became shorter than 1 μm after 6 h (Figure 4B). After 12 h, most primary cilia (stained green by acetylated α‐tubulin) disappeared, and only the basal body (stained red by γ‐tubulin) could be seen. The basal body also became smaller and dimer gradually (Figure 4A). Meanwhile, the proportion of cells with primary cilia changed in a similar tendency, which was 90% originally but decreased to 10% after 12 h (Figure 4C). These changes coincided with the decreases in protein expression levels of acetylated α‐tubulin and γ‐tubulin as revealed by Western blotting analyses (Figure 4D,E). However, the protein expression levels of α‐tubulin and GAPDH kept constant throughout the treatment periods, demonstrating that the above changes induced by microgravity were specific and not lethal to the cells. The above‐described changes did not happen in the control cells without RPM treatment.

### 3.4 Microgravity‐induced reduction in the expression of miRNA‐129‐3p was related to the cilium resorption

**FIGURE 5:**
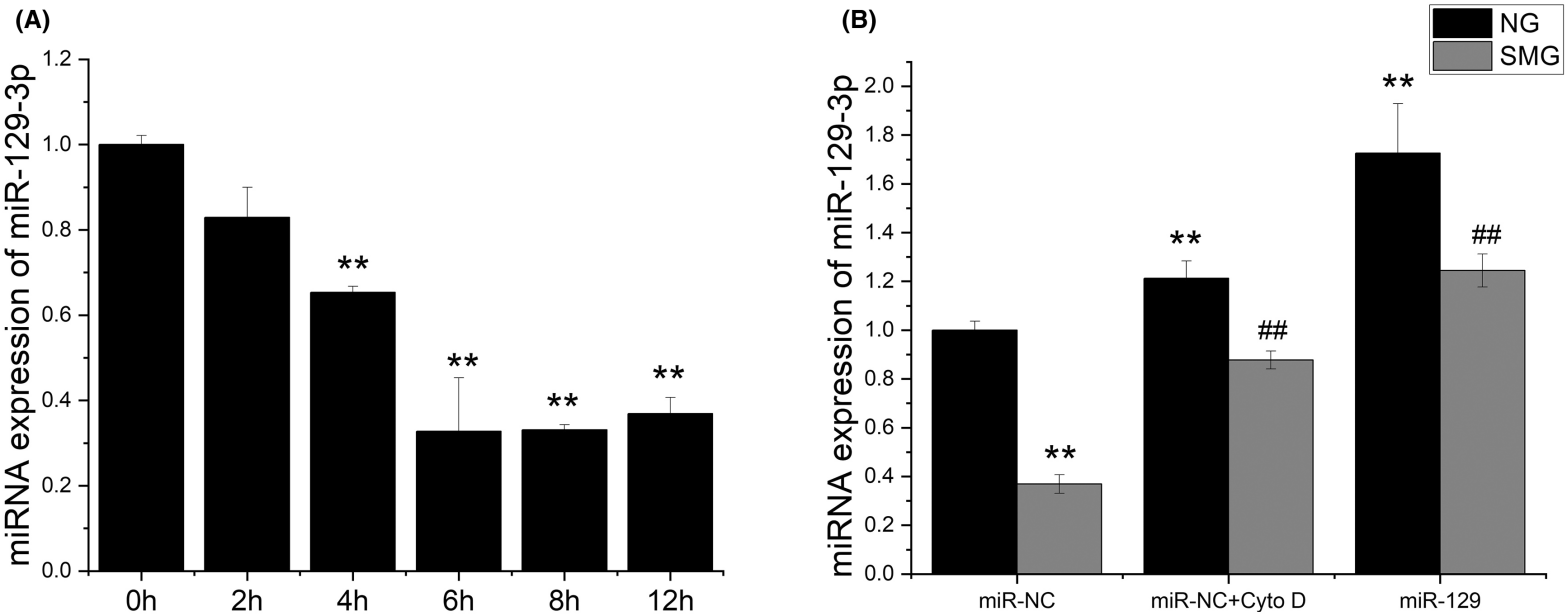
Changes in the expression levels of miRNA‐129‐3p (miR‐129) of rat calvarial osteoblasts (ROBs) when exposed to random positioning machine (RPM)‐induced microgravity and treated by cytochalasin D (Cyto D). (A) The miRNA‐129‐3p expression levels after different periods of RPM treatment. (B) The miRNA‐129‐3p expression levels after 12 h of RPM treatment in the cells pretreated by 0.1 μM cytochalasin D or with miRNA‐129‐3p overexpression. NG: normal gravity; SMG: simulated microgravity. **p < 0.01 vs 0 h in NG condition; ^##^ *p* < 0.01 vs the negative control group (miR‐NC) in SMG condition.

It was reported that blocking expression of a microRNA, miRNA‐129‐3p, inhibited serum‐starvation‐induced ciliogenesis and miRNA‐129‐3p inhibition in zebrafish embryos suppressed ciliation in Kupffer's vesicle and induced developmental abnormalities.^32^ Enlightened by this report, we decided to find out whether the expression level of miRNA‐129‐3p in osteoblasts is affected by microgravity. ROBs were exposed to RPM‐simulated microgravity for 0, 2, 4, 6, 8 or 12 h, respectively, and then were subjected to real‐time RT‐PCR analyses for expression levels of miRNA‐129‐3p. It was found that the mRNA expression level of miRNA‐129‐3p decreased significantly after 4 h of RPM treatment (*p* < 0.01), and further and persistently decreased to one‐third of the original level starting from 6 h (Figure 5A). No such changes occurred in the control cells without RPM treatment. These results indicated that microgravity‐induced resorption of primary cilia may be related to the decreased expression of miRNA‐129‐3p. To verify this possibility, we examined the expression levels of miRNA‐129‐3p in the ROBs exposed to microgravity and pretreated by cytochalasin D, a metabolite derived from moulds and reported to prevent microgravity‐induced resorption of primary cilia.^29^ As expected, the expression level of miRNA‐129‐3p in cytochalasin D treatment group (miR‐NC + Cyto D) was significantly higher than the control group (miR‐NC) in both normal gravity (NG) condition and SMG condition (*p* < 0.01). Although the expression level of miRNA‐129‐3p in miRNA‐129‐3p overexpression group (miR‐129) was significantly lower in SMG condition than in NG group (*p* < 0.01), it was significantly higher than that of the miR‐NC group in SMG condition (*p* < 0.01) (Figure 5B). These results suggested that microgravity‐induced resorption of primary cilia should be caused by the reduction of the miRNA‐129‐3p expression level.

### 3.5 Microgravity‐induced resorption of primary cilia can be prevented by overexpression of miRNA‐129‐3p

**FIGURE 6:**
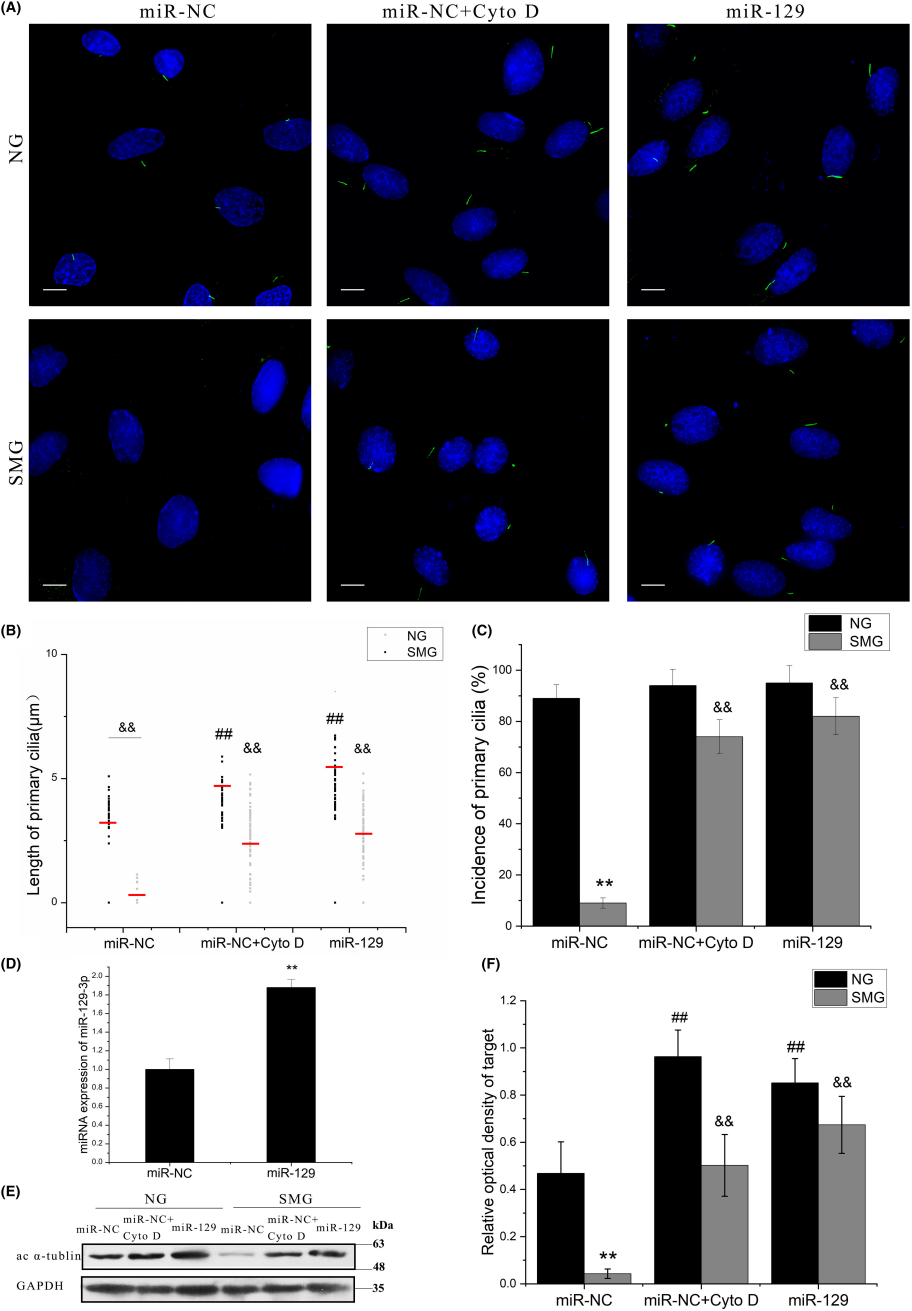
Effects of treatment with cytochalasin D (Cyto D) or miRNA‐129‐3p (miR‐129) on the length and incidence of primary cilia of rat calvarial osteoblasts exposed to random positioning machine (RPM)‐induced microgravity. (A) The morphology of primary cilia in each group after 12 h of exposure to RPM treatment. (B) The means (red bar) and individual measurements of cilium length in the normal gravity group (NG) and the simulated microgravity group (SMG). (C) The percent of cells with primary cilia. (D) Comparison of miRNA‐129‐3p mRNA expression levels between the negative control group (miR‐NC) and the miRNA group (miR‐129) after 24 h of transfection. (E‐F) Representative Western blots demonstrate the expression levels of acetylated α‐tubulin (ac α‐tubulin). NG, the control group in normal gravity; SMG, the group in simulated microgravity; miR‐NC, the negative control group; miR‐NC + CytoD: the negative control group with cytochalasin D treatment; miR‐129, the group with miRNA‐129‐3p overexpression. Each experiment was conducted at least three times independently. Scale bar = 10 μm. **p < 0.01 vs miR‐NC group; ^##^ *p* < 0.01 vs the negative control group (miR‐NC) in the NG condition; ^&&^ *p* < 0.01 vs the negative control group (miR‐NC) in the SMG condition.

Since the expression level of miRNA‐129‐3p was reduced significantly and Cao et al. reported that the overexpression of miRNA‐129‐3p promoted cilium biogenesis,^32^ the present study investigated whether overexpression of this miRNA can prevent microgravity‐induced loss of primary cilia. As shown in Figure 6, when miRNA‐129‐3p was overexpressed in ROBs exposed to simulated microgravity (Figure 6D), both the resorption of primary cilia and the RPM treatment‐induced reduction in the proportion of ROBs with primary cilia were significantly attenuated when compared to the control group (miR‐NC). In addition, despite statistical insignificance, miRNA‐129‐3p overexpression (miR‐129) appeared a little more efficient than cytochalasin D treatment (miR‐NC + CytoD) in keeping the length and incidence of primary cilia in ROBs (Figure 6A–C). Consistently, the changes in the expression levels of acetylated α‐tubulin among three groups displayed a similar tendency (Figure 6E,F).

### 3.6 Protection of primary cilia by miRNA‐129‐3p overexpression prevented microgravity‐induced loss of osteogenic potential of ROBs

**FIGURE 7:**
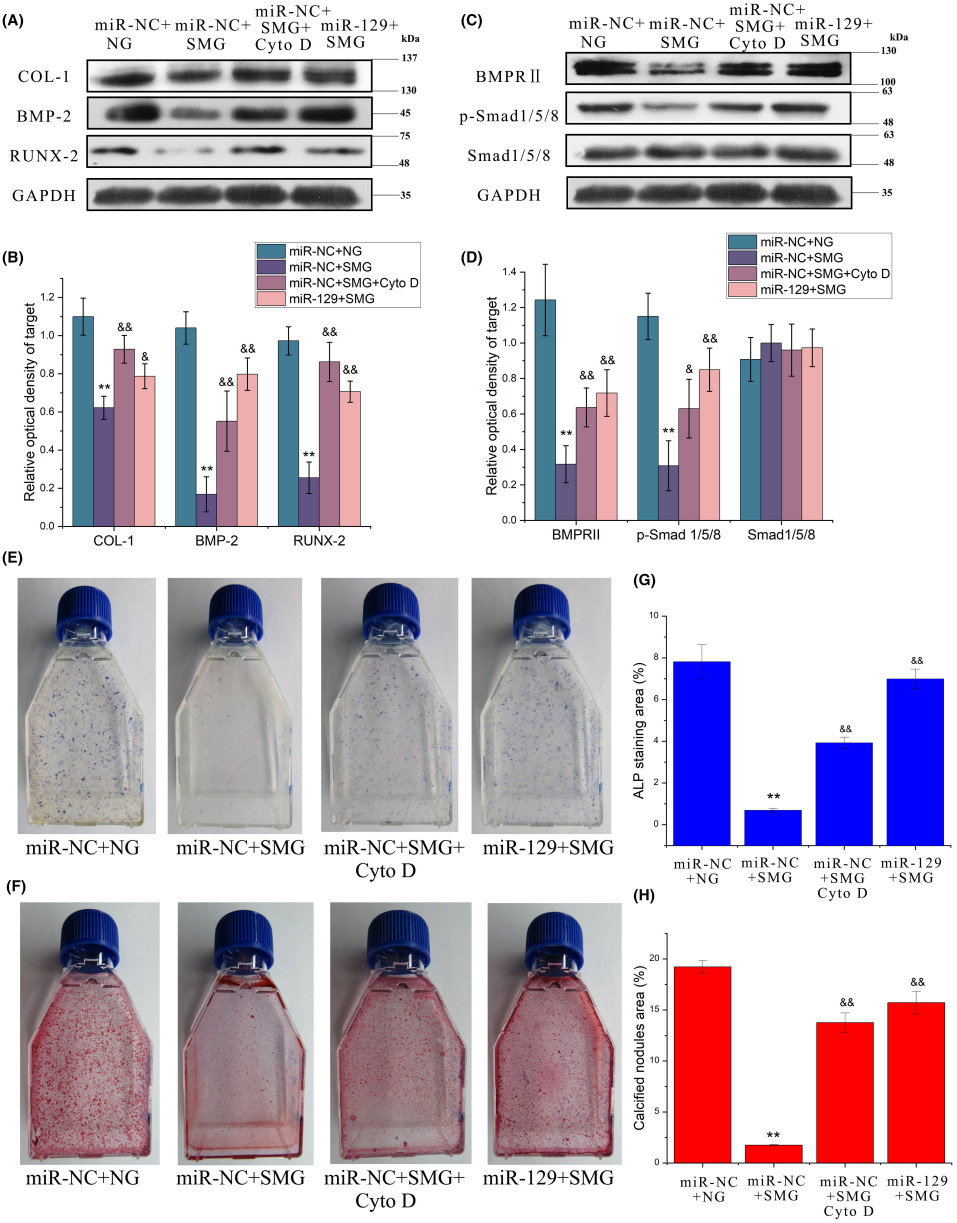
Overexpression of miR‐129‐3p exerts similar protective effects with cytochalasin D treatment on the osteogenic potential and activation of the BMP‐2/Smad1/5/8 signalling pathway in rat calvarial osteoblasts exposed to random positioning machine (RPM)‐induced microgravity. (A, B) Representative Western blots demonstrate the protein expression levels of the osteogenic markers including COL‐1, BMP‐2 and Runx‐2. (C, D) Representative Western blots demonstrate the protein expression levels of the BMP‐2/Smad1/5/8 signalling pathway components, including BMP‐2, p‐Smad1/5/8 and Smad1/5/8. (E) Representative images and the quantitative areas (F) of ALP‐stained CFU‐F‐ALP colonies after 9 days. (G) Representative images and the quantitative areas (H) of the Alizarin red‐stained mineralized nodules after 12 days. miR‐NC + NG: the negative control group in normal gravity; miR‐NC + SMG: the negative control group in simulated microgravity; miR‐NC + CytoD + SMG: the negative control group with cytochalasin D treatment in simulated microgravity; miR‐129 + SMG: the group with miRNA‐129‐3p overexpression in simulated microgravity. **p < 0.01 vs miR‐NC + NG group; ^&^ *p* < 0.05, ^&^ *p* < 0.01 vs miR‐NC + SMG group.

Since the overexpression of miRNA‐129‐3p in ROBs prevented RPM‐induced abrogation of primary cilia, we wondered whether it could retain the osteogenic potential of osteoblasts as well as cytochalasin D (a positive control) in simulated microgravity. As shown in Figure 7A,B, protein expression levels of three osteogenesis markers (COL‐1, BMP‐2 and RUNX‐2) were significantly reduced in the miR‐NC + SMG group compared with the miR‐NC + NG group but were kept at similar levels with those of the miR‐NC + NG group in the miR‐129 + SMG group or miR‐NC + CytoD + SMG group. Consistently, the CFU‐F‐ALP colonies formed after 9 days (Figure 7E) and the calcified nodules formed after 12 days (Figure 7F) displayed a similar tendency among these groups, and the areas of CFU‐F‐ALP colonies and calcified nodules in the miR‐129 + SMG group were bigger than those of the miR‐NC + CytoD + SMG group (*p* < 0.01). Moreover, as judged by the expression levels of BMPRII and p‐Smad1/5/8 (Figure 7C,D), activation of the BMP‐2/Smad1/5/8 signalling pathway in the miR‐129 + SMG group and miR‐NC + CytoD + SMG group was maintained at a similar level as with the miR‐NC + NG group.

**FIGURE 8:**
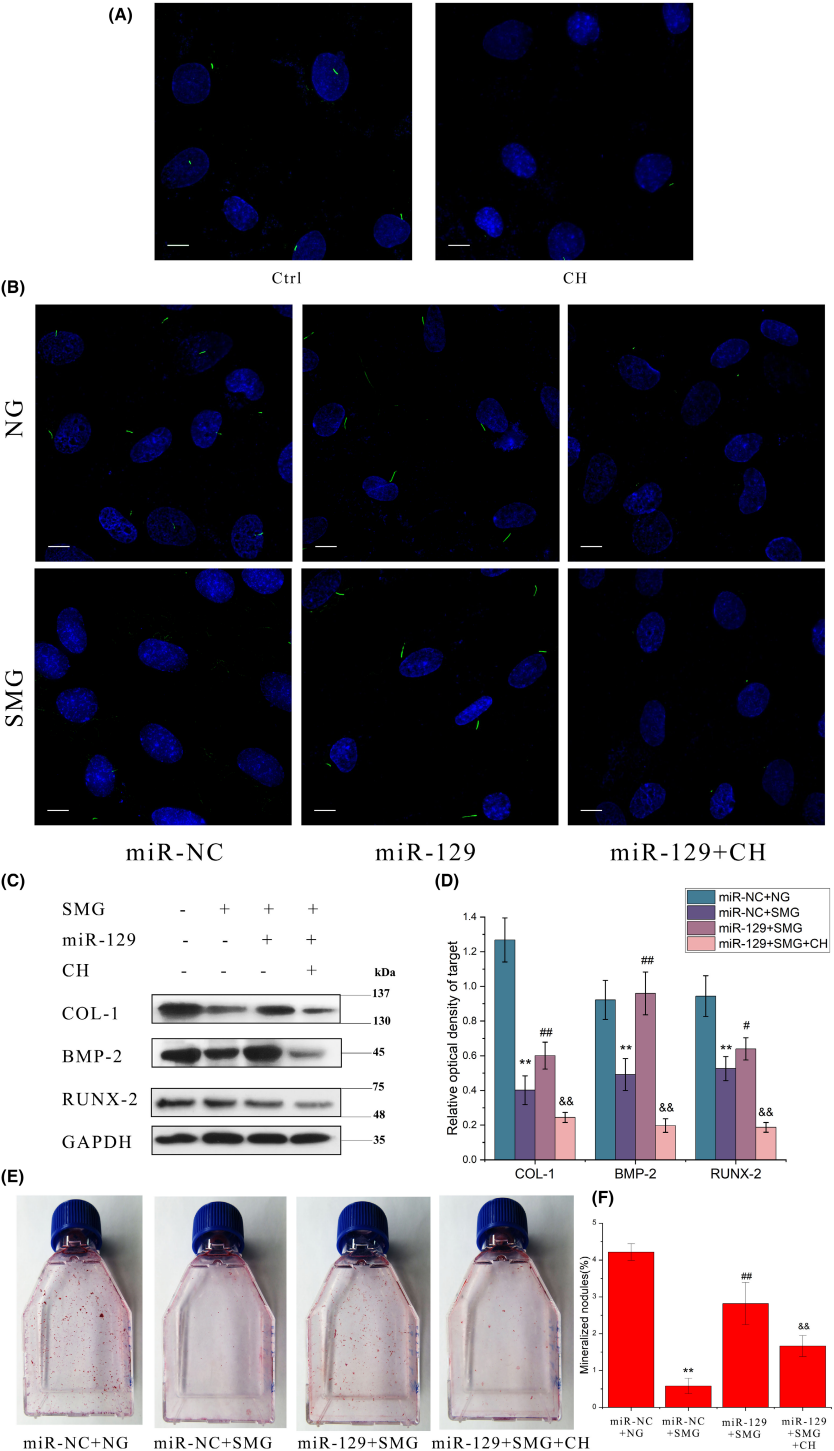
Effect on primary cilia and the osteogenic potential of rat calvarial osteoblasts treated with miRNA‐129‐3p and exposed to random positioning machine (RPM)‐induced microgravity. (A) Comparison between the cells treated with chloral hydrate (CH) or solvent vehicle control (Ctrl). (B) The morphology of primary cilia in different conditions. (C, D) Representative Western blots demonstrate the protein expression levels of osteogenic markers including COL‐1, BMP‐2 and Runx‐2. (E) Representative images and quantitative areas (F) of the Alizarin red‐stained mineralized nodules after 12 d. NG: normal gravity; SMG: simulated microgravity; miR‐NC + NG: the negative control group in normal gravity; miR‐NC + SMG: the negative control group in simulated microgravity; miR‐129 + SMG: the group with miRNA‐129‐3p overexpression in simulated microgravity; miR‐129 + SMG + CH: the group with miRNA‐129‐3p overexpression and chloral hydrate treatment in simulated microgravity. Scale bar = 10 μm. **p < 0.01 vs miR‐NC + NG group; ^##^ *p* < 0.01 vs miR‐NC + SMG group; ^&&^ *p* < 0.01 vs miR‐129 + NG group.

To further verify if miRNA‐129‐3p‐induced attenuation of the loss of osteogenic potential of osteoblasts was due to its protection of primary cilia, we removed the primary cilia with chloral hydrate (CH)^33^ before examining the preventive effect of miRNA‐129‐3p on ROBs. As shown in Figure 8A, 4 mM CH removed the primary cilia of ROBs clearly, and the removing effect was still effective in the cells with miRNA‐129‐3p overexpression (Figure 8B). When the cells in the miR‐129 + CH group were exposed to RPM treatment (miR‐129 + CH + SMG group), protein expression levels of the osteogenesis markers (including COL‐1, BMP‐2 and RUNX‐2) were decreased greatly compared with those of the miR‐129 + SMG group, and were near to those of the miR‐NC + SMG group (Figure 8C,D). Consistently, the numbers of calcified nodules formed after 12 days displayed similar tendencies among the four groups (Figure 8E,F). These results indicated that preventive effect of miRNA‐129‐3p overexpression on the osteogenic potential of osteoblasts exposed to microgravity requires the functionality and integrity of primary cilia.

## 4 DISCUSSION

Many studies have demonstrated that living in microgravity results in reduced bone formation due to impairments in osteoblastic formation and function,^34^, ^35^ for which the underlying mechanism is still unclear. Since Russian investigators found reductions in some blood antioxidants and an increase in lipid peroxidation in humans after long‐term space flights, oxidative stress has been proposed to be one of the reasons for microgravity‐induced bone loss, which led to the recommendation for adequate intake of antioxidants that had been proved to be only partially effective.^34^, ^35^, ^36^, ^37^, ^38^ In 2017, we reported that the decreased osteogenesis of osteoblasts in simulated microgravity was accompanied by the shortening/resorption of primary cilia.^29^ The current study has further found that the microgravity‐induced loss of osteoblast function and shortening of primary cilia were related to the decreased activity of BMP2 signalling, and that the overexpression of miRNA‐129‐3p, a microRNA found downregulated in microgravity‐treated osteoblasts, could counteract the adverse effect of microgravity on primary cilia and the osteogenic capacity of osteoblasts.

Due to the critical roles of BMPs in skeletal development, bone formation and bone repair,^39^ some studies have focused on changes in BMP signalling in microgravity and on its potential use as a therapeutic means.^17^ Cao et al. demonstrated the declined expression of BMPs and transforming growth factor‐beta (TGF‐β) in all regions of the hind‐limb bones of rats with tail suspension for 21 days.^40^ Another study showed that BMP‐2 lost its ability to stimulate the osteogenic differentiation of ROS17/2.8 cells under simulated weightlessness.^41^ Qian et al. reported that interval intramuscular injection of BMP2 alone in hindlimb‐unloaded rats could increase the osteogenic potential of bone marrow‐derived stromal cells and the expression levels of osteogenic marker genes.^42^ Despite the above studies, the detailed mechanism for microgravity‐induced decreased activity of BMP signalling is still unknown.

We were the first group to report that the primary cilia of osteoblasts became shorter quickly after exposure to RPM‐simulated microgravity, which was accompanied by a significant reduction in osteogenic differentiation and mineralization.^29^ We speculated that primary cilia may be a microgravity sensor of osteoblasts and that keeping the normal structure and function of primary cilia should be a potential way to prevent the loss of osteoblast function induced by microgravity. Supporting this, cytochalasin D, an inhibitor of actin polymerization was found to prevent microgravity‐induced loss of osteogenic capacity of osteoblasts by protecting primary cilia.^29^, ^30^ In the current study, it was further found that microgravity‐induced shortening/resorption of primary cilia was accompanied by the significant reduction in the expression levels of signalling proteins of the BMP‐2/Smad15/8 signalling pathway including BMP2, BMPRII, BMPRIB and p‐Smad1/5/8 (Figure 2), indicating the inhibited status of BMP2 signalling pathway. Moreover, it was found that BMP2, BMPRII, Smad1/5/8 and p‐Smad1/5/8 were all found to be localized to the primary cilia of osteoblasts, and exogenous recombinant BMP2 lost its ability to stimulate the osteogenic differentiation and mineralization of the osteoblasts that had the primary cilia being abrogated by silencing the expression of IFT88. These results suggest the following sequence of events in the pathophysiological process of microgravity‐induced osteogenesis defect: the primary cilia of osteoblasts are firstly resorbed due to microgravity, and activation of the BMP2/Smad15/8 signalling pathway (and perhaps other pathways) is then inhibited, which in turn results in the impairment of osteogenic potential of osteoblasts. This process can suggest that the protection of primary cilia should be the primary means to prevent the microgravity‐induced impairment of osteoblasts.

miRNA‐129‐3p is a microRNA conserved in vertebrates that was found to be able to control cellular cilium biogenesis by downregulating centrosomal protein (CP110) and concomitantly repressing the formation of branched F‐actin.^32^ The serum starvation‐induced ciliogenesis in RPE1 cells has been attributed to the increased expression of miRNA‐129‐3p.^32^ Inspired by this report, we examined the expression levels of miRNA‐129‐3p in the ROBs exposed to simulated microgravity in a time course. Surprisingly, it was found that the changing tendency of miRNA‐129‐3p expression was very similar to those of the length and incidence of primary cilia in treated ROBs (Figures 4 and 5). This result, together with the simultaneous increase in miRNA‐129‐3p expression in the cells treated by cytochalasin D, demonstrated that the reduction in miRNA‐129‐3p expression could be the main reason for microgravity‐induced resorption of primary cilia.

To further verify the role of miRNA‐129‐3p in microgravity‐induced resorption of primary cilia and to explore its potential therapeutic effect, we next performed a simulated microgravity experiment with ROBs with miRNA‐129‐3p overexpression (transfected with a plasmid containing miRNA‐129‐3p). Compared with the control group, in the group with miRNA‐129‐3p overexpression, microgravity‐induced reductions in the length and incidence of primary cilia were both significantly attenuated, and the impaired osteogenic differentiation as well as the inhibited activity of the BMP‐2/Smad15/8 signalling pathway were also almost rescued completely (Figures 6 and 7). Furthermore, this protective effect of miRNA‐129‐3p overexpression did not occur in the osteoblasts in which primary cilia had been removed by chloral hydrate treatment (Figure 8), excluding the possibility that the protective effect was through a non‐cilium mechanism. Consistent with our findings, Cao et al. had also microinjected fertilized zebrafish eggs with 129‐MO, an antisense morpholino oligonucleotide against miRNA‐129, and found that the average number and length of cilia in Kupffer's vesicle were reduced by 50.5% and 37.3%, respectively, which lead to 71.0%–87.7% of the embryos or larvae exhibiting a curved body and pericardial oedema at 72 h post‐fertilization.^32^ Our findings indicated the important role of miRNA‐129‐3p in microgravity‐induced resorption of primary cilia and its potential as a therapeutic target to protect primary cilia and thus osteoblast function in microgravity.

In conclusion, the current study revealed for the first time that exposure of the osteoblasts to microgravity downregulates the expression of miRNA‐129‐3p, which in turn causes the resorption of the primary cilia and the inhibition of the BMP‐2/Smad15/8 signalling pathway, and consequently leads to the impairments of osteoblast formation and function. Our results indicated that the protection of the primary cilia resulting from miRNA‐129‐3p treatment could be an effective countermeasure against the impairment of osteoblasts induced by microgravity. However, further studies are needed to verify this in vitro result in the situation of bone loss in hindlimb‐suspended rodents or even human beings in microgravity and to investigate whether it is the case in other types of cells and tissues.

## AUTHOR CONTRIBUTIONS

**Jing Liu:** Data curation (lead); writing – original draft (equal). **Fei‐Fan Leng:** Data curation (equal). **Yu‐Hai Gao:** Data curation (equal). **Wen‐Fang He:** Data curation (equal). **Ju‐Fang Wang:** Methodology (equal). **Xian J. Cory:** Writing – review and editing (equal). **Hui‐Ping Ma:** Data curation (equal); writing – review and editing (equal). **Ke‐Ming Chen:** Conceptualization (lead).

## FUNDING INFORMATION

National Natural Sciences Foundation of China (81770879, 31870851); Youth Project of Gansu Science Foundation (20JR5RA589).

## CONFLICT OF INTEREST

The authors declare that they have no conflict of interest.

## Supporting information


AppendixS1

## Data Availability

The datasets used and/or analysed during the current study are available from the corresponding author on reasonable request.
