# Supplementary material for: Protection of primary cilia is an effective countermeasure against the impairment of osteoblast function induced by simulated microgravity
Source: J Cell Mol Med. 2022 Dec 13;27(1):36–51. doi: 10.1111/jcmm.17628 (PMC9806295; doi:10.1111/jcmm.17628)
Supplement: Supplementary file 1 — AppendixS1 [file JCMM-27-36-s001.docx]

Supplementary Figure 1. The ratio of colocalized signal cells in cilia. Percentage of cells with colocalization signal with cilia in all cells.
